# Unravelling Functional Neurology: an overview of all published documents by FR Carrick, including a critical review of research articles on its effect or benefit

**DOI:** 10.1186/s12998-019-0287-2

**Published:** 2020-01-28

**Authors:** Marine Demortier, Charlotte Leboeuf-Yde

**Affiliations:** 1 CIAMS, University of Paris-Sud, University of Paris-Saclay, F-91405 Orsay Cedex, France; 2 CIAMS, University of Orléans, F- 45067 Orléans, France; 3 Institut Franco Européen de Chiropraxie, 24 boulevard Paul Vaillant Couturier, F- 94200 Ivry sur Seine, France; 4 Institute for Regional Health Research, University of Southern Denmark, DK-5000 Odense, Denmark

**Keywords:** Functional Neurology, Chiropractic, Critical review, Evidence, Effect, Benefit

## Abstract

**Background:**

Functional Neurology (FN), founded by FR Carrick, is an approach used by some chiropractors to treat a multitude of conditions via the nervous system including the brain. However, it seems to lack easily obtainable scientific evidence for its clinical validity.

**Objectives:**

1) To define the topics of FR Carrick’s publications, 2) to define the proportion of articles that are research studies, case studies, abstracts and conference papers, 3) to define how many of these are clinical research studies that purported or appeared to deal with the effect or benefit of FN, 4) in these studies, to establish whether the design and overall study method were suitable for research into the effect or benefit of FN, and 5) to describe the evidence available in relation to the clinical effect or benefit of FN, taking into account seven minimal methodological criteria.

**Method:**

A literature search was done on *Pubmed* from its inception till October 2018, supplemented by a search on *Scopus* and *ResearchGate* to find all published documents by FR Carrick. We identified their types and topics, retaining for a critical review full text scientific articles appearing to test effect/benefit of FN procedures, subjecting them to a basic quality assessment (scoring 0–7). Results from studies of methodologically acceptable standard would be taken into account.

**Results:**

We found 121 published texts, 39 of which were full scientific research articles. Of these, 23 dealt with topics relating to FN. Fourteen articles reported on clinical validity but only seven included a control group. The methodological quality of these seven articles was low, ranging between 1.5–4 out of 7. We therefore did not further report the outcomes of these studies.

**Conclusion:**

We found no acceptable evidence in favour of effect/benefit of the FN approach. We therefore do not recommend its promotion as an evidence-based method. Further research on this topic should be conducted in collaboration with independent scientific institutions using commonly accepted research methods.

**Trial registration:**

**PROSPERO** This review was registered in PROSPERO (application date 23.02.2019; no CRD42019126345).

## Résumé

**Introduction:**

La Neurologie Fonctionnelle (NF), telle que fondée par FR Carrick, est une approche thérapeutique principalement utilisée par certains chiropracteurs. Elle permettrait de traiter de nombreuses conditions via le système nerveux, notamment, en stimulant le cerveau. Cependant, l’évidence scientifique à propos de sa validité clinique est difficilement accessible.

**Objectifs:**

1) définir les thèmes de publications de FR Carrick, 2) définir la proportion d’articles de recherche, d’études de cas, de résumés et de documents de conférences, 3) parmi eux, dénombrer les études de recherche clinique qui examinent/semblent examiner l’effet ou le bénéfice d’un traitement en NF, 4) définir si le schéma et la méthode de ces études sont adaptés pour étudier l’effet ou le bénéfice de la NF, 5) décrire l’évidence scientifique disponible concernant l’effet ou le bénéfice de la NF en prenant en compte sept critères méthodologiques basiques.

**Méthode:**

Une recherche bibliographique a été effectuée jusqu’en octobre 2018 sur *Pubmed*, *Scopus* et *ResearchGate* pour recueillir l’ensemble des documents publiés par FR Carrick et identifier leurs types et leurs thèmes respectifs. La qualité méthodologique des articles disponibles en texte intégral et semblant tester l’effet/le bénéfice d’une approche en NF a été évaluée pour établir leurs validités cliniques (score de 0 à 7). Seuls les résultats des études, ayant une méthodologie en accord avec les standards de recherche actuels ont été retenus.

**Résultats:**

121 documents publiés par FR Carrick ont été recueillis, dont 39 en texte intégral. 23/39 articles s’intéressent à un thème associé à la NF. Quatorze d’entre eux, examinent la validité clinique de la NF, et seulement sept études ont inclus un groupe contrôle. La qualité méthodologique de ces sept études est faible, variant de 1.5 à 4/7. Les résultats de ces études n’ont donc pas été rapportés.

**Conclusion:**

Il semble donc qu’il n’y ait pas d’évidence scientifique en faveur de l’effet ou du bénéfice d’une intervention en NF. La NF ne devrait donc pas être promue comme une approche fondée sur les faits. Enfin, toutes nouvelles recherches sur ce sujet devraient être menées en collaboration avec des institutions scientifiques indépendantes et adopter une méthodologie en accord avec les standards de recherche actuels.

## Introduction

Functional Neurology (FN) is a relatively recent therapeutic approach, founded in 1979 by a Canadian chiropractor, Frederick Robert Carrick (FRC) [1]. FN is based on the theory that lesions in the nervous system, including the brain, consisting of groups of dysfunctional neurons, explain many health conditions and that these lesions can be successfully improved by various types of stimulation, including spinal manipulation [2]. According to a previous scoping review on FN, it is claimed that FN can be used to treat numerous varied conditions, such as musculoskeletal, neurodevelopmental, and neurodegenerative disorders [2]. FN is, therefore, anticipated to be potentially useful to many people who suffer from pain and dysfunction, often of a chronic type [2].

FN is also attractive for some practitioners, perhaps because this non-invasive treatment aims at the ‘cause’ within the nervous system, without much need to deal extensively with the typical pathophysiology of the disease itself. This characteristic can be assumed, as practitioners, who are taught to use FN, do not, as a rule, receive hospital training within the various specialties, a point found in the course descriptions available on the Carrick Institute website [3]. The FN technique is, for example, popular with some chiropractors, with 13% of Australian chiropractors, who participated in a recent survey, reporting that they use it [4].

It is possible to obtain extensive training in FN, including at a professional training center in Florida established by FRC, known as the Carrick Institute. FRC also provides seminars all over the world, as is described on his website and through active publicity via the internet [1, 3]. According to this website, more than 14,000 persons have been trained in this concept [1].

By composition, FN seems to be based on a combination of material from many areas, covering central neuro-anatomy, neurophysiology, various types of neuro-rehabilitation and includes various ‘rules’ for when and how spinal manipulation shall be undertaken [2]. It appears complicated and necessitates considerable knowledge of the central nervous system. The FN seminars provided by the Carrick Institute concentrate heavily on this topic [3]. Since FN is taught mainly at private seminars, it is relatively difficult to obtain objective information in the public domain about its rationale, scientific basis, and clinical validity.

In a recent scoping review, it was established that one reason why it was difficult to find scientific literature on this topic, was possibly because the label “FN” is not necessarily used in the title or text of such articles [2]. A subsequent critical review was therefore undertaken of a journal, *Functional Neurology, Rehabilitation, and Ergonomics*, that specializes in this topic and was recommended in personal correspondence by its editor-in-chief, Gerry Leishman, who regarded this journal as a suitable source of scientific information on FN, as explained in that review [5]. The aim of that journal review was to investigate the evidence for clinical benefit or effect of FN. The conclusions drawn were that, although 36 research articles were published in its 24 issues (edited between 2011 and 2016), this journal contained no methodologically sound studies on the clinical benefit or effect of FN [5].

Nevertheless, the authors of that review acknowledged that valuable information could still have been published elsewhere. To partly overcome the problematic keyword search and to add an important element to a comprehensive review of the topic that touches many areas, it was suggested that a reasonable and potentially useful approach would be to review all publications authored by the founder of FN, FRC [5]. FRC states that his work is based on research [6]. Further, an initial cursory search demonstrated that he appears to publish extensively. Therefore, it was anticipated that he had produced some pertinent and useful research to support claims of the effects/benefits of FN. On the other hand, FRC’s apparent starting point as a researcher in FN, an article on how spinal manipulation could affect the size of the blind spot in the eye [7], resulted in a lively debate through letters to the editor [8–18]. For us, it seems fair to say that FN is a controversial subject. The burden of evidence would therefore exceed discussions on the anatomy, physiology and pathology of the nervous system and case-reports purportedly proving the link between treatment and a positive outcome and instead requires clinical effect studies of good quality.

The purpose of this critical review was, therefore, to examine an important element of the evidence available regarding FN, being that produced by the founder of FN, FR Carrick on the effect/benefit of FN treatment. The specific objectives were:

1. To define the topics of FR Carrick’s publications
2. To define the proportion of articles that are research studies, case studies, abstracts and conference papers.
3. To define how many of these are clinical research studies that purported or appeared to deal with the effect or benefit of FN.
4. In these studies, to establish whether the design and overall study method were suitable for research into the effect or benefit of FN.
5. To describe the evidence available in relation to the clinical effect or benefit of FN, taking into account seven minimal methodological criteria.

## Methods

### Search for published documents

We searched the *PubMed* database from its inception until October 2018 supplemented by searches in *Scopus* and *ResearchGate* for publications by Frederick Robert Carrick. Our research equation was: “Carrick FR” OR “Carrick F” OR “Carrick, Frederick” OR “Carrick, Frederick Robert” OR “Carrick, Frederick R”. Answers to the editor found in Scopus were not included. In some instances, the same article appeared twice on *ResearchGate*; all such duplicates were excluded. However, when the same title appeared twice, for example both as an article and as an abstract from a conference, both were included. We consulted also the webpages of the Carrick Institute for information on publications. All obtained publications were listed in a table (Additional file 1), described by title, year of publication, journal, topic, source (*PubMed, Scopus,* or *ResearchGate*), and type of study design. In addition, we noted if they consisted of full-texts or not.

### Classification into topics

First, using information in Additional file 1, based on the title and the abstract (if available), we sorted all documents into four main topics: i) *Brain, ii) Posture or/and Balance, iii) Other Functional Neurology,* and *iv) Other Non-Functional Neurology* topics*.* The topic ‘*Brain’* included titles and abstracts including the following words: “brain”, “mental”, “post-concussion syndrome”, “stroke”, “cognitive”, “post-concussive”, “schizophrenia”, “paranoia”, “vegetative state”, “paralysis”, “PTSD”, “palsy”, “aphasia”, “diplopia”, “nystagmus”, “paraplegia”, “blepherospasm”, “cerebellar activity”, “tremor”, “memory”, “comatose”, “brainstem”, “autistic spectrum disorder”, “coma”, and “ADD/ADHD”. The topic ‘*Posture/Balance’* included titles and abstracts with the words “posturography”, “vertigo”, “balance”, “vestibular”, “ataxia”, and “posturographic”. If terms from ‘*Brain*’ and another topic were included, we classified the document under *‘Brain’*. The topic ‘*Other FN’* included titles and abstracts dealing with neurological symptoms but not obviously with brain or posture/balance, but topics nevertheless thought of us to be typically dealt with in Functional Neurology (e.g: “multimodal neurorehabilitation” and “peripheral somatosensory stimulation”). Anything else was included in ‘*Other Non-FN’,* i.e. documents that did not relate to Functional Neurology. Examples of ‘*Other Non-FN*’ are titles such as “Colorectal Cancer Risk Awareness and Screening Uptake among Adults in the United Arab Emirates” and “The Federation of Student Islamic Societies Programme to Challenge Mental Health Stigma in Muslim Communities in England: The Fosis Birmingham Study”.

### Selection of research documents

Second, we tried to obtain all documents in full text, but when this was not possible, we used only the abstract, if available. Based on full texts or abstracts, we attempted to determine the research design of each document, as we were searching for research articles. However, when we had access only to titles, we excluded them from this classification process.

Articles were considered by us to be ‘research studies’, when they had some type of research question or when it was possible to interpret the background text in such a way, and/or if there was an obvious methods section. In some instances, we asked for and obtained full texts via *ResearchGate*. Research designs were roughly divided into Surveys/Hospital records, Clinical studies, and Experimental studies. Studies were classified as ‘clinical’, if the text related to patients, diseases, or treatments, whereas they were classified as ‘experimental’, if it was clear or probable that study subjects were asymptomatic or that some technical aspect, instrument, or method was tested without a clear clinical goal. We also identified case reports/case series and discussion papers/letters to editor/ editorial/thesis, although we did not consider these as research papers. If the design was unclear, we reported it as ‘unclear’. Full texts were reported separately from abstracts only, to obtain an overview of the type and depth of research activities that had been published. Please see Additional files 2a and b.

### Selection of research articles on effect/benefit of treatment/intervention using the Functional Neurology approach

Third, we scrutinized the full text of research articles for words that indicated that they dealt with effect or benefit of intervention/treatment on topics relating to FN. For this, we searched for words such as “effectiveness”, “effect”, “improvement”, “recovery”, and “efficacy”.

### Selection of research articles appearing to study effect/benefit of treatment/intervention using the Functional Neurology approach and using correct study design

The fourth step was to retain, for our review, full text articles that appeared to investigate effect/benefit of treatment/intervention, that is, if the study design made it possible to do so. We therefore selected full text articles, if they fulfilled one of these two basic criteria: i) ‘Effect’ studies should, as a minimum, compare an intervention group to a placebo/sham treated group or, possibly, the intervention group should be compared to a valid control group, in which treatment previously has been shown to be superior to placebo/sham. ii) Benefit studies should compare the intervention group to some other type of treatment or to untreated controls. When this was not fulfilled, we provided a brief explanation of the design problem and, although listed in a table (Table 1), they were not included in the review.

**Table 1:** Table showing whether studies used an appropriate or potentially appropriate study design to investigate effect or benefit of treatment

First author Journal Year of publication Reference number	Condition studied	Was design appropriate to investigate effect or benefit of intervention?	If design was not appropriate, major methodological considerations
Carrick FR, et al. Fontiers in Neurology 2018 [31]	Autism Spectrum Disorders in children	Yes	
Carrick FR, et al. Frontiers in Neurology 2017 [22]	Chronic post concussion	No	No control group
Noone P, et al. Biomedical Sciences Instrumentation 2017 [28]	Posture		Not appropriate for this review. This was in fact a pilot study to investigate a method to measure posture was robust enough to use in different data collection settings and to allow polling of data
Carrick FR, et al. Frontiers in Neurology 2016 [30]	Stroke	Yes	
Carrick FR, et al. Frontiers in Public Health 2015 [24]	Post Traumatic Stress Disorder in Veterans (PTSD)	No	No control group
Carrick FR, et al. Frontiers in Public Health 2015 [25]	Post Traumatic Stress Disorder in Veterans	No	No control group
Pagnacco G, et al. Biomedical Sciences Instrumentation 2015 [29]	Balance performance	Yes, probably	
Carrick FR, et al. Biomedical Sciences Instrumentation 2015 [23]	Vestibular problems	No	No control group
Carrick FR, et al. Functional Neurology Rehabilitation and Ergonomics. 2011 [26]	Postural balance	No	No control group
Carrick FR, et al. Functional Neurology Rehabilitation and Ergonomics 2013 [20]	Balance stability	Yes, probably	
Daubeny N, et al. International Journal on Disability and Human Development 2010 [21]	Brain function	Yes	
Leisman G, et al. International Journal of Adolescent Medicine and Health 2010 [27]	Attention deficit disorder (ADD) and Attention Deficit Hyperactivity Disorder (ADHD) in children	No	No control group
Carrick FR, The Journal of Alternative and Complementary Medicine 2007 [32]	Posture	Yes	
Carrick FR Phase II Journal of Manipulative and Physiological Therapeutics 1997 [7]	Brain Function	Yes	

### Description of articles selected for review

The reviewed articles have been briefly described in Table 2 including information about ethics approval, trial registration, conflict of interest, and funding.

**Table 2:** Description of seven articles authored or co-authored by FR Carrick reporting on effect or benefit of the Functional Neurology approach and using an appropriate study design

1st Author [Ref] Year Journal	Condition studied	Study subjects : - Type - Age (range) - Origin - Number (analyzed/included)	- Intervention - Control (other than sham) - Sham	- Outcome variables	When was it assessed ?	- Ethics approval (with a clear mention of its origin) - Trial registration	- Conflict of Interest (CoI) (reported or supposed) - Funding
Carrick [31] 2018 Front Neurol	Autism Spectrum Disorder (ASD)	- ASD children - 4-17 years -? - 34/83	- Mente Autism Device - NA - Sham Device	- qEEG - ASD questionnaires - Posturography - a) Delta-alpha ratio - b) Power ratio index - c) Brain symmetry index (a,b and c collected through qEEG) - NIH Stroke Scale	- Before - After 12 weeks	- « our Institutional Review Board » - NCT02773303	- Reported no CoI - Carrick Institute - Plasticity Brain Center - Neurotech International Limited
Carrick[30] 2016 Front Neurol	Stroke	- Adults with acute middle cerebral artery ischemic stroke - > 39 years - “our intensive care unit” - 34/34	- Aspirine + 3 repetitions of saccades/ smooth pursuits, 3 treatments a day - Aspirine - NA		- Before, upon admission - After 7 days of treatment	- “our Institution” - No	- Reported no CoI - Carrick Institute - Plasticity Brain Center
Pagnacco [29] 2015 Biomed Sci Instrum	Postural Balance	- Adults - 46 - Normal subjects − 39/?	- Toned based stimulation - own control -?	- Stability score	- Suring the experiment	- Yes “the Carrick Institute for Graduate Study IRB#1 - Neurology” - No	- Not reported - Not reported
Carrick [20] 2013 Funct Neurol Rehabil Ergon	Postural Balance	Study 1: - Healthy Adults - 20-60 years -Volunteers through advertisements - 52/52 Study 2: - Healthy adults - 20-61 years - Volunteers through advertisements - 56/56	Both studies: - Whole body rotation over 40s for all groups - Each group (4 groups per study) differed in terms of pitch and yaw planes during whole body rotation - NA	Study 1: 8 posturographic measures Study 2: 6 posturographic measures	Both studies: - Before - Immediately after - After 1 day - After 1 week	- “our IRB” - NA	- Not reported - Not reported
Daubeny [21] 2010 Int J Disabil Hum Dev	Brain Function	- Healthy adults -? -? - 62/ 62	- Upper extremity manipulations - NA - Upper extremity manipulation with unloaded activator	- Blind-spot size	- Before - Immediately after	- No - No	- Reported no CoI but at least 2 authors are known to have business interest in relation to the topic. - No
Carrick [32] 2007 J Altern Complement Med	Postural Balance	- Adult healthy volunteers - 22-74 years -? - 266/270 (210 + 60)	- 9 groups of listening to different types of music (several interventions, probably also serving as controls) - White noise (unclear if considered as sham)	- Force platform (CAPS score)	- Before - After 10 min - After 1 week - After 1 month	- “Our institutional review board” - NCT00121693	- Reported no CoI, but reported that two authors owned two patents on the posturographic device used in the study. - Carrick Institute
Carrick [7] 1997 J Manipulative Physiol Ther	Brain Function	Phase II: - Adult volunteers -? - enrolled in various postdoctoral programs - 20	(Unusual design, study subjects requiring ‘correct’ treatment were either treated ‘correctly’ or ‘incorrectly’) - Manipulation of C2 at the ‘correct’ side - Manipulation of C2 at the ‘incorrect’ side	- Blind spot size	- Before - After	- No - NA (not possible/common at this time)	- Not reported - Not reported

### Critical review of articles that used a study design able to deal with effect/benefit of treatment/intervention using the Functional Neurology approach

To review these articles, we used a slightly modified approach, as previously reported in a critical review of a journal specialized in information relating to FN [5]. In summary, all reviewed articles were reported in tables for description and quality and further summarized narratively. An observation in that previous review [5] was that the methodological quality of studies was generally weak, so we considered it reasonable to perform only a basic examination of the methods sections in the included articles. We considered quality, based on selected risk-of-bias items from the Cochrane recommendations [19] and two additional quality items relating to external validity. We gave one point for each correct approach and sometimes 0.5 for partially correct items. Ultimately, we used a seven items quality checklist (Table 3) as explained below.

**Table 3:** Studies authored or co-authored by FR Carrick that potentially would be suitable to report on effect or benefit of treatment: Sources of bias and methodological quality (method section)

-Title of study -Journal -Yr of publication	Effect studies: Were study subjects shown to be blind to treatment (1 point), stated to be blind (0.5 point) Benefice studies: Were study subject stated to be naïve to treatment (1 point) /1 point	-Was a random allocation reported? (0.5 point) -Was it stated that it was concealed? (NA if no random allocation) (0.5 point) /1point	Were interventions well described? /1 point	Was the assessor reported to be blind on all outcome measurement? /1 point	Outcome measurement reported to be reliable or reproducible? /1 point	Was the person who analyzed the data stated to be blind? /1 point	Were losses and exclusion reported or obvious in results, tables or graphs? /1 point	Total score /7
The treatment of Autism Spectrum Disorder with Auditory Neurofeedback: A randomized Placebo Controlled Trial Using the Mente Autism Device. Front Neurol 2018	- No - No	-Yes - No	- Yes	Q-aire: NA Posturography No qEEG: No	- Q-aires: Yes -Posturography: Unclear *(“… proven* [23] *to exceed the metrological characteristics for clinical posturography recommended by the International Society for Posture and Gait Research* [24]*.”* - qEEG: No	- No	- Yes	43%
	0/1	0.5/1	1/1	0/1	0.5/1	0/1	1/1	3/7
Eye-Movement Training Results in Changes in qEEG and NIH Stroke Scale in Subjects suffering from acute middle cerebral artery ischemic stroke: a randomized control trial Fron Neurol 2016	- NA - No	- Yes - Yes	- Yes	- Q-aire: NA - qEEG: No	(Introduction) - qEEG: No - National Institute of Health Stroke Scale: No	- No	- Yes	43%
	0/1	1/1	1/1	0/1	0/1	0/1	1/1	3/7
Effect of tone-based sound stimulation on balance performance of normal subjects: preliminary investigation Biomed Sci Instrum 2015	Benefit study - No	- No - NA	- perhaps	- No	- Yes	- No	- No	29%
	0/1	0/1	1/1	0/1	1/1	0/1	0/1	2/7
The effect of off vertical axis and multiplanar vestibular rotational stimulation on balance stability and limits od stability Funct Neurol Rehabil Ergon 2013	- NA - No	- Yes - No	- Yes	- No	- No	- No	- No	21%
	0/1	0.5/1	1/1	0/1	0/1	0/1	0/1	1.5/7
Effects of contralateral extremity manipulation on brain function Int J Disabil Hum Dev 2010	- No	- Yes - No	- Yes	- Yes	- No	- No	- Yes	50%
	0/1	0.5/1	1/1	1/1	0/1	0/1	1/1	3.5/7
Posturographic Changes Associated with Music Listening J Altern Complement Med 2007	Benefit study - No	- Yes - No	- Partially (duration of exposure?)	- No	Yes (Introduction)	No	Yes, but we could not verify the losses. Nevertheless, 0.5 point given as a benefit of doubt	36%
	0/1	0.5/1	0.5/1	0/1	1/1	0/1	0.5/1	2.5/7
Changes in Brain after Manipulation of cervical spine (Phase 2) J Manipulative Physiol Ther 1997	- NA - No (from CI)	- No- NA	- Yes	- Yes	- Yes	- No	Yes	57%
	0/1	0/1	1/1	1/1	1/1	0/1	1/1	4/7

*Seven methodological review items*

1. In “effect studies” we asked whether study subjects had been *shown* to be blinded to the type of treatment (1 point) or, at least, *stated* to have been blinded (0.5 point) to avoid expectation bias. In studies of “benefit of treatment/intervention”, we expected the study subjects to be naïve to the study type or expected study outcome, also to avoid expectation bias (1 point).
2. To avoid selection bias, we expected studies to have allocated their subjects randomly (0.5 point) and to have done this in a concealed fashion (0.5 point), resulting in 1 point if both criteria were reported. However, we did not deem it necessary to assess which type of allocation was used.
3. We expected the intervention to be well described, which would be necessary to give transparency, ability to replicate, and to ensure external validity (1 point).
4. To avoid expectation bias or conscious manipulation of the data collection, the assessor should be reported to have been blinded on the link between intervention group and outcome measurements, which gave 1 point.
5. Ideally, all outcome variables used should be reported reliable or reproducible (1 point). If this information was missing for some variables, the presence of at least one reliable or reproducible variable gave 0.5 point. Imprecise or unsuitable outcome measurements can result in errors in any direction and errors in this domain are therefore not necessarily bias-related but refer to lack of methodological quality in general, including external validity.
6. Expectation bias may also occur if the person who analyses the data can link the participants to their specific allocation group, for which reason it should be stated that the analysis was done blindly (1 point).
7. Loss of participants or data points can result in attrition bias, which occurs if the persons who disappear from the study or data points that are not included in the analysis are related primarily to one group or if these persons tended towards one type of outcome. Therefore, losses and exclusions should be declared or be obvious in results, tables or graphs. If imputation of missing data was used, then this should be described. Further, results in text and tables should concur (1 point if there was clear reporting and data transparent).

### Data extraction

Two of our articles [20, 21] had been previously reviewed [5] but we nevertheless reviewed them again. Information was extracted from the articles by the two authors, independently, and unclear items discussed. A third person could be called upon in case of disagreement. However, there was no need to discuss disagreements relating to the judgement of the quality items. Although not pre-planned, we asked the advice of other researchers to confirm our interpretation of some of the texts, when we found them confusing. This will be indicated in the Result section.

### Interpretation of data

To interpret our quality checklist table, we created a score based on the number of points, using the same approach as in a previous review on this topic [5]. For ease of interpretation we transformed this score into percentages. Each study was then described in terms of quality assessment. The quality scores were used to give an impression of the general methodological quality of the reviewed articles. Thus, a score of 1/7 indicates a total lack of methodological awareness, whereas a score of 7/7 would not necessarily indicate a ‘perfect’ study. Because the quality checklist was somewhat basic, we expected studies to approach full scores to qualify as providing valid information, and the results would be reported for methodologically acceptable studies only.

## Results

**Fig. 1:**
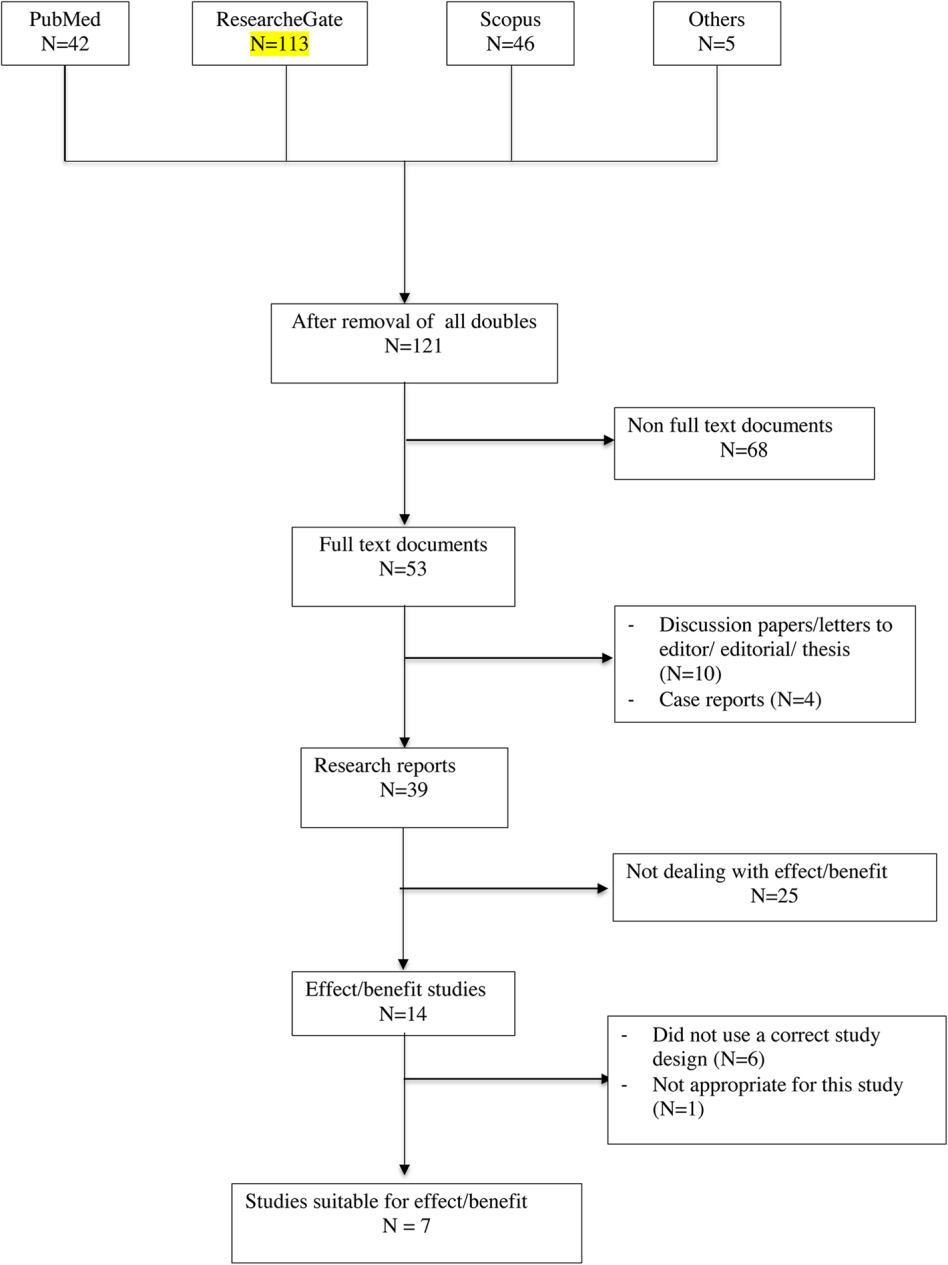
Description of the search of literature for effect/benefit studies published by FR Carrick

We found 121 documents containing the name of Carrick (as described in the methods) somewhere in the list of authors obtained from PubMed (*N* = 42), Scopus (*N* = 46), and ResearchGate (*N* = 110). In addition, five articles were found from other sources. Many documents were identified in all three sources but ResearchGate contained more titles than the others. For detailed information, please see the flow chart (Fig.1) and Additional file 1.

### Topics of publications

These documents were classified into the following topics based on titles and abstracts. ‘*Brain’* (*N* = 70), ‘*Posture or/and Balance*’ (*N* = 24), ‘*Other Functional Neurology* ‘(N=6), and ‘*Other Non-Functional Neurology*’ (N=21) topics*.* Some titles (identical or near-identical) appeared several times (N=5), e.g. both as an article and as an abstract for some sort of ‘event’. For a list of documents by topic see Additional file 1.

### Proportion of articles that are research studies, case studies, abstracts and conference papers

**Fig. 2:**
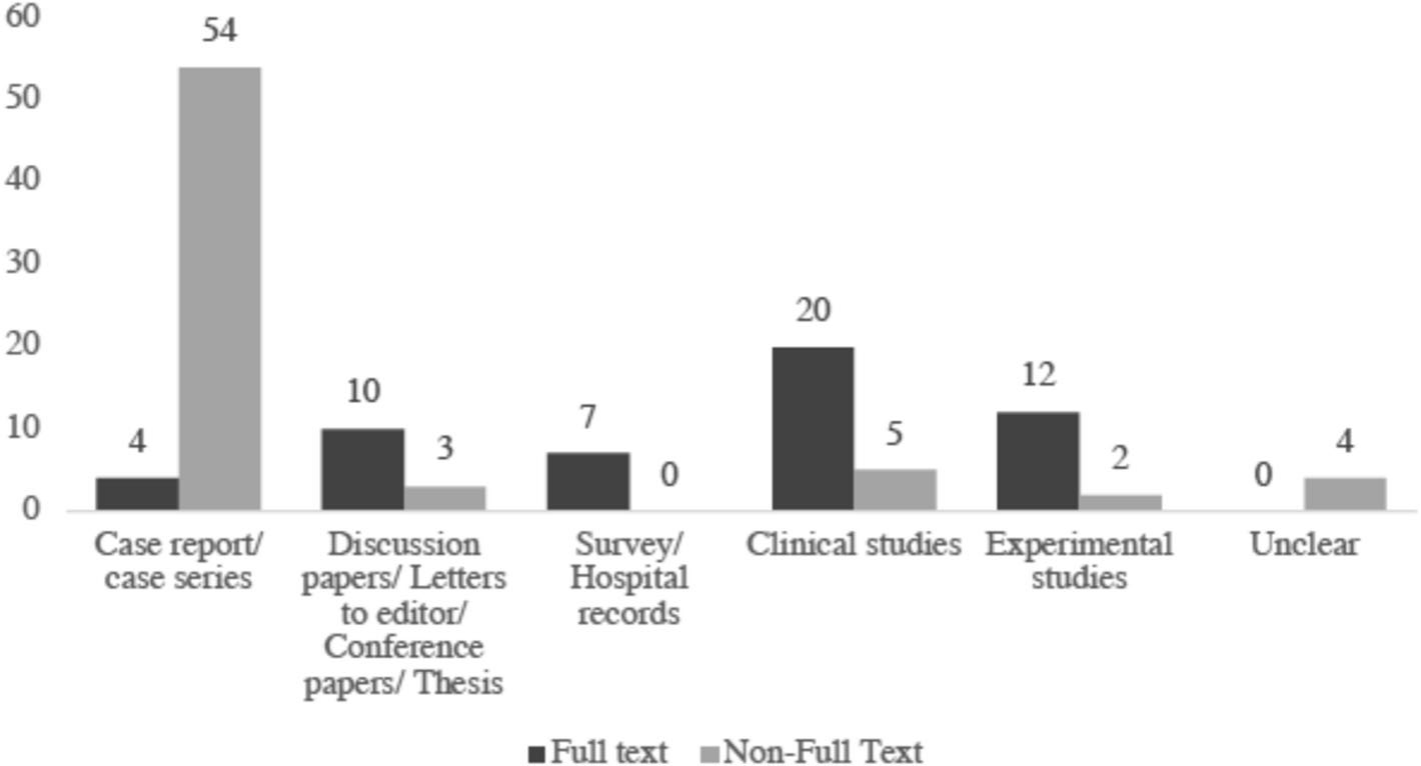
The number of published texts authored or co-authored by FR Carrick shown by type of publication and whether full text or non-full text documents

The number and types of research documents have been shown in Additional file 2a (full text *n* = 53) and 2b (non-full text *n* = 68), separating those that we could obtain as full text articles from those that we identified only as abstract texts (Fig. 2). Among the full text articles, the two most common topics were ‘*Brain*’ (*N* = 20) and ‘*Other Non-Functional Neurology*’ (*N* = 19); mostly classified by us as being clinical studies. In the non-full texts, the topic ‘*Brain*’ was the most common (*N* = 50), reported mainly as single case reports or as case series without an experimental design.

### Studies purporting to study effect/benefit of treatment/intervention

Fourteen full text research articles appeared to deal with effect or benefit of a treatment or intervention using elements of Functional Neurology. They were selected because they used words such as “effect”, “changes”, “beneficial impact” (Table 1).

### Studies on effect/benefit with a control group

Thus, fourteen articles appeared to have studied the effect/benefit of treatment/intervention. However, six [22–27] of these were removed, because there were no control groups and could therefore only report on ‘outcome’, which may or may not have been caused by the treatment/intervention. Another article [28] was later removed, because, on further scrutiny, it was found not to be appropriate for this review. In fact, this was a pilot study to investigate if a method to measure posture was robust enough to use in different data collection settings and to allow pooling of data.

### Methodological assessment of seven studies on effect/benefit with a control group

#### Description

The remaining seven articles are described in Table 2. In brief, they reported on the treatment of several conditions, namely: abnormal brain function, autism spectrum disorder, and stroke. They also studied balance and posture.

The treatment/intervention consisted of various sounds [29], manipulation (cervical or extremity) [7, 21], or ocular movements [30]. For example, a computer-based auditory software program, the Mente Autism Device, was used to treat autism spectrum disorders [31], and eye movements were used to treat acute middle cerebral artery ischemic stroke [30]. Posture and balance were influenced during body rotation [20] and also, with different types of music [32]. Brain function was evaluated using the “blind spot” and stimulated using cervical manipulation [7].

As can be seen in Table 2, col. 7, ethics approval was mainly given by “own institutional review board” [33], which we interpreted as the Carrick Institute. Two of the trials had been registered in a trials register (ClinicalTrials.gov) (Table 2, col. 7, 2nd and 7th rows). Conflict of interest was reported in four studies (Table 2, col.8). The Carrick Institute was reported to have funded three studies (Table 2, col.8, rows 2, 3 and 7).

#### Quality assessment

These remaining seven full text articles (Table 3) lacked important aspects of scientific rigor, with scores ranging from 1.5 / 7 (21%) to 4/7 (57%). Thus, five of these seven articles scored between 21 and 43% and the remaining two scored 50 and 57%, respectively (Table 3). Typically, subjects were not blind or naïve to treatment, some outcome measurements were not stated to be reliable nor reproducible, and usually the assessor and statistician were not reported to be blind.

For example, the study scoring 21% (1.5/7) had reported that there was random allocation but did not state if it was concealed. The only other fulfilled quality item of the required seven was ‘interventions well described’. The study that scored 57% (4/7) failed to report if study subjects were naïve to treatment, if there was a random allocation into treatment groups, and if the person analysing the data was blind to treatment group. A brief description of each article is provided below, with articles sorted in descending order by methodological quality score.

### “Changes in Brain Function after Manipulation of the Cervical Spine” [7]

This article from 1997 [7] (score 4/7; 57%) is the first scientific report on FN published by FRC, therefore possibly considered as the original scientific basis of FN. In this article, FRC tested the hypothesis that brain activity can change as a consequence of spinal manipulation, as detected by observed changes in the size of the ‘blind spot’ of the eye. The study is, in the abstract, described as a “large (500 subjects) double-blind controlled study”, in which 12 hypotheses were tested on various subgroups of these 500 people. We included in our review the “phase 2 procedure”, described in the study, in which twenty subjects with predetermined identified increased ‘blind spot’ findings were either subjected to the ‘correct’ treatment (i.e. manipulation of C2 on the same side as the enlarged cortical map) or the ‘incorrect’ treatment (i.e. manipulation of C2 on the opposite side of the enlarged cortical map). With the ‘correct’ treatment, the ‘blind spot’ was reported to have normalized in size, whereas this did not happen with the ‘incorrect’ treatment, in accordance with the theory.

A review of the method revealed that allocation to treatment was not reported to have been determined in a random fashion. The ‘blind spot’ was apparently measured subjectively, without optometric equipment, but had been “confirmed” by two examiners, both at base-line and follow-up. It was unclear what the label ‘double-blind’ referred to, as only the examiners were clearly reported to be blinded. It could be speculated that the study subjects were uninformed of the purpose of the study and perhaps the blind spot changes could not be affected by expectation bias but this was not stated in the paper. Study subjects were said to have been enrolled in ‘post-neurology programs at a variety of institutions’, hence possibly chiropractors, who may or may not have been naïve to the aims of the study. In addition, the definition of an enlarged blind spot was not provided. The result tables indicated that some type of continuous variable had been used, since t-tests were reported to have been used to test for differences between groups. It is therefore likely that the circumference or area of the blind spot was measured but, if so, it was not detailed. It was also not clear who undertook the statistical analysis and whether it was done without that person knowing which treatment was provided.

We did not include in this review the other tests reported in this study, in which the remaining study subjects (*N* = 480) were included, because they did not compare different treatment groups but tested other types of hypotheses. Conflict of interest, funding, and human research ethics committee approval were not reported, but this was not common at that time.

### “Effects of contralateral extremity manipulation on brain function” [21]

In this study [21] (score 3.5/7; 50%), 2 × 31 healthy adults received either an upper extremity manipulation or a sham manipulation with an unloaded activator instrument in a random fashion. The blind spot size was estimated ‘manually’ before and immediately after intervention and found to have changed in a pre-hypothesized manner. This article was scrutinized in a previous review [5], in which it was noted that, although this is a randomized controlled trial, the reliability/reproducibility of the blind spot was uncertain, (ii) the study subjects were not described, and (iii) the statistical analysis was not reported to have been blinded. Hence methodological issues could potentially affect the validity of the results. Furthermore, ethics committee approval, trials registration, and conflict of interest were not reported.

### “The Treatment of Autism Spectrum Disorder with Auditory Neurofeedback: Randomized Placebo Controlled Trial Using the Mente Autism Device” [31]

In this study [31] (score 3/7; 43%), 83 subjects, previously diagnosed as having an autistic spectrum disorder, were randomized into two groups: one intervention group (active) and one placebo/sham group. The treatment consisted of 12 weeks of home-based Neurofeedback therapy delivered by the Mente Autism therapy device, which produces binaural beats in the ears of the participants. These sounds were selected according to the child’s individual EEG pattern recorded by the device. The control group used an identical device, but the binaural beats where randomly generated. Outcome variables were 1) qEEG, which was defined as “statistical analysis of EEG” and stated to “allow highly precise measurement of brain activity and connectivity”, 2) dynamic computerized posturography, and 3) five autism spectrum disorder questionnaires (Autism Treatment Evaluation Checklist, Social Responsiveness Scale-Second Edition, the Behaviour Rating Inventory of Executive Function, the Autism Behaviour Checklist, and the Questions About Behavioural Function). There were two evaluations, one at enrolment and the second after 12 weeks of treatment. In total, 49/83 (59%) subjects dropped out and the statistical analysis included only 34 subjects.

Methodological problems were that the subjects were not shown to be blind or naïve, the randomisation was not stated to be concealed, the assessor and the person who analysed the data were not stated to be blind, and two of the outcome variables (qEEG and posturography) were not stated to be reliable and reproducible.

We noted that the study was funded by the Carrick Institute, the Plasticity Brain Center and Neurotech International Limited. It was reported that authors had no conflict of interest. This study was registered in ClinicalTrials.gov and was stated to have been approved by “own institutional review board”.

### “Eye-Movement Training Results in Changes in qEEG and NIH Stroke Scale in Subject Suffering from Acute Middle Cerebral Artery Ischemic Stroke: A Randomized Control Trial” [30]

In this study [30], scoring 3/7; 43%, 34 subjects with non-disabling ischemic middle cerebral artery stroke were randomly divided into two groups using a computer program. The control group (*n* = 17) received the standard treatment (aspirin) and the treatment group (n = 17) received the standard treatment plus eye-movement training. It is possible that the study took place in a hospital in Cuba (assumption based on the acknowledgements) but this was not explicitly stated in the main text. The results were measured after one week with a stroke scale (the National Institute of Health Stroke Scale - NIHSS) and a visualization of brain function (qEEG). Significant differences in favour of the intervention group were reported for the qEEG but not for the stroke scale, although the title indicates that this was the case also for the stroke scale.

The major methodological problem in this study is that the qEEG was not reported as reliable and reproducible. In fact, the qEEG does not appear to be an easily quantified outcome variable, as it involves various computer-generated colours appearing in different parts of the brain, requiring an objective and reproducible measurement method and an understanding of what the different areas relate to and whether they are pertinent in the treatment of stroke. The study was described by the authors as “double-blind”, but as there is no sham intervention, only a control group (treated with aspirin), control subjects cannot have been blind to the type of treatment, which usually is the case when studies are described as ‘double-blind’. Descriptions of the other types of blinding are not given. Therefore, it is not possible to determine which types of bias could have affected the results. When there is no sham treatment given in the control group, and blinding is impossible, study subjects should instead be naïve to the treatment method, to avoid expectation bias, which is a good idea to prevent their post-treatment performance to be boosted through expectations. However, this was not reported.

The study was, to our knowledge, not reported in a trial registry prior to performance, so it was not possible to establish, if it was conducted according to the original plan and ethics permission was provided by “our Institution”; however, it is unknown what this refers to, the Carrick Institute or the (possible) Cuban hospital. According to the report, there were plans to perform a one-year follow-up the study. As this was published in January 2016, and the time of writing this present report is August 2019, it appears not to have been done. Conflict of interest was not reported but funding was stated to have been provided by the Carrick Institute and the Plasticity Brain Center.

### “Effect of tone-based sound stimulation on balance performance of normal subjects: preliminary investigation” [29]

In this study [29] (score 2/7; 29%), thirty-nine subjects were subjected to various sounds and their balance tested on an unstable surface. Study subjects were said to be their own control and the outcome test was reported reproducible. The article includes a detailed explanation of the intervention (sound) but we found it more difficult to understand the study design, except that interventions were not provided randomly, which would be relevant, as we expect there would be a learning curve for balance. An explanation of how to combine variables was lacking; therefore we found it difficult to understand how variables were combined in the report. This is a preliminary study, and there is also a study on balance/ posturogaphic changes and music listening with a larger study sample (next summary).

### “Posturographic Changes Associated with Music Listening” [32]

In this study [32] (score 2.5/7; 36%), 210 healthy volunteers were randomly divided into four groups, of which three listened to different types of music: i) Mozart, ii) a slow song of Nolwenn Leroy, or iii) a fast song of Nolwenn Leroy, and iv) one control group (who listened to ‘white noise’). Thereafter, another 60 healthy volunteers were included to listen to six other artists, resulting in 10 groups. Balance was studied using a force platform, before and after intervention, using a comprehensive assessment, with subjects having closed eyes and standing on a perturbing surface. This was tested after 10 min, 1 week and 1 month. In all, 35 (13%) of the subjects dropped out. Individual scores were held up against values that were said to be normative in relation to age groups. Outcome was reported in tables as numbers and percentages of subjects who changed to move into the ‘normal’ ranges.

Some music was reported to improve postural stability more than white noise. Methodologically, it would have been advantageous if the report had described the study subjects’ characteristics and whether they were naïve or not, the duration of the intervention, and whether the assessor and statistician were blinded to treatment group. Unfortunately, the numbers of losses reported in the table and text were not easily identified.

No conflict of interest was reported but it was “funded by the Carrick Institute”. The study was registered in ClinicalTrials.gov and authorized by their “own institutional review board”.

### “The Effect of Off Vertical Axis and Multiplanar Vestibular Rotational Stimulation on Balance Stability and Limits of Stability” [20]

This study [20], previously reviewed [5] and re-reviewed by us, dealt with postural reactions to various positional interventions (1.5/7; 21%) and has been reported and discussed in some detail in a previous review [5]. It seems to be a description of a randomized controlled trial with four different subgroups receiving different interventions, but it is also possible that it is an outcome study simply observing these four groups. Numerous tests took place on only few study subjects and, apart from random allocation having been reported, a thorough explanation of the intervention procedure, and clearly reported losses and exclusion, the other methodological checklist items were unsatisfactory. Conflict of interest and funding were not reported. The ethics committee was their own “IRB” (abbreviation for Institutional Review Board) and there was no trial registration, although, if this was a simple outcome study rather than a randomized study, this would not be necessary. In sum, this was considered both a confusing and methodologically weak study. In the previous review [5] assistance had been needed from a methodologist to try to understand the study design, and this person was called in to re-verify the interpretation of the long and dense method section.

#### Summary of results

Because the methodological approach in the seven reviewed research articles had a low quality score, none exceeding 57%, we did not have confidence in the validity of the results and have not dealt with these.

## Discussion

### Summary of findings

This study is a critical review of all sourced documents published by FR Carrick, who is stated to be the founder of the Functional Neurology approach. We found 121 publications, of which 100 related to this topic. Of these, 1/3 only were full texts, and, of the others, 80% consisted of case-reports. Among the 39 full text publications on FN, 14 dealt with the benefit or effect of FN but only 7 compared treatment or intervention with a control group. We considered the methodological quality to be low, for which reason the results are, in our opinion, not suitable for further elaboration.

### General comments

These observations were similar to those noted in a previous review on FN [5], which dealt with articles on FN from a journal specialized in this topic, i.e. relatively many published texts, few research articles, and poor-quality methodology [5]. Further, our general observation was that the texts sometimes are dense and confusing. This makes it difficult for the reader to grasp the essential aspects of the research projects and to trust their results.

However, it is of course still possible that FN is an effective therapy. Therefore, in view of the potential benefits of FN on a multitude of chronic and poorly understood conditions, it is important that it is tested in methodologically sound studies, reports published in peer-reviewed journals, and that any positive findings are reproduced preferably by specialist independent research teams.

### Methodological considerations of own work

To search for literature on FN is challenging, as a method of treatment without clear limitations cannot easily be identified with the usual PICO approach. Also, as has been stated previously, relevant literature cannot be found when searching simply with the terms ‘Functional Neurology’ [6]. FRC is quoted with the following words regarding FN: “There is more evidence than one can ever read in a life-time” [6]. For these reasons, we wanted to complement the previous review of articles in the journal: *Functional Neurology, Rehabilitation and Ergonomics* [5] by screening all scientific work by the central researcher, FRC himself.

To access all articles authored or co-authored by FRC, we searched *Pubmed*, *ResearchGate*, and *Scopus*, in the hope of finding more relevant research. Additional scrutiny of introduction and discussion texts plus reference lists of included articles failed to reveal any undetected publications. However, when we consulted the website of the Carrick Institute to see if they were any publications of which we were unaware, we found another three texts.

Our critical review was based on well accepted concepts of bias and validity of information and has been used previously [5]. We were temperate and minimalistic in our quality checklist. For example, we did not check the validity of outcome variables but trusted the author if they were said to be valid with a reference. Nor did we consider whether the sample size was adequate.

In view of the low quality scores, we could of course have used various quality items as extra inclusion/exclusion criteria. However, the use of a more stringent inclusion/exclusion approach would not have been an advantage, as it would likely have resulted in fewer articles for the review. Our inclusive approach (excluding only studies without a control group) gave us the opportunity to explain the design and methodological issues to readers, including clinicians, who are not always familiar with critical literature reading.

The review was performed independently by the two authors, who compared findings and solved most problems through discussion, but we sometimes required the help of others. Thus we had to ask advice on two articles [20, 31], from a person with extensive experience in reading texts on FN and from an experienced epidemiologist/methodologist. Also they found the methods and result sections difficult to interpret.

For us, the most difficult study was on ‘pitch and yaw’ [20], which was so complicated to interpret that we failed to grasp what type of research design had been used (a randomized controlled trial or an outcome study with multiple subgroups). It is, therefore, possible that we may have misinterpreted some information. Although this would be unfortunate, it is important for authors and editors to ensure that the text of all articles is understandable for both researchers and general clinician readers.

### Perspectives

On a *policy level*, in view of the lack of evidence on the effect/benefit of Functional Neurology as taught by FRC, we currently do not recommend that it should be promoted as evidence based.

On a *clinical level*, clinicians should be aware that, presently, there appears to be no (relatively easily) available evidence on the effect or benefit of Functional Neurology, as taught by FRC. To justify the use of a method without sound scientific clinical evidence could be problematic for regulated practitioners.

Finally, on a *research level*, absence of evidence of effect is not the same as evidence of absence of effect. On the other hand, all ideas and concepts may not be worthwhile to study but if further research is undertaken on this topic, it would be useful if the studies were conducted in collaboration with independent scientific institutions using commonly accepted research methods.

## Conclusions

We reviewed FR Carrick’s published Functional Neurology research over the last approximately 20 years. In that time, he has published what corresponds to approximately six texts per year. Based on our review, only seven of these could be used to study the effect/benefit of the treatment/intervention of Functional Neurology, and, because we found these articles methodologically weak, we did not feel that we could draw any conclusions on its clinical validity based on FRC’s work.

## Supplementary information

**Additional file 1:** A list of published texts authored or co-authored by FR Carrick obtained on PubMed, Scopus or ResearchGate, shown by study topic, study design and whether full text or not full text documents.

**Additional file 2: **a: The number of published *full text documents* authored or co-authored by FR Carrick shown by type of publication (*N* = 53).b: The number of published *non full text documents* authored or co-authored by FR Carrick shown by type of publication (*N* = 68).

## Abbreviations

ADD: Attention Deficit Disorder
ADHD: Attention Deficit Hyperactivity Disorder
CI: Carrick Institute
CoI: Conflict of Interest
EEG: ElectroEncephalGram
FN: Functional Neurology
FRC: Frederick Robert Carrick
NIHS: National Institute of Health Stroke Scale
PTSD: Post Traumatic Syndrome Disorder
qEEG: Quantitative Electroencephalography
