# Supplementary material for: Unravelling Functional Neurology: an overview of all published documents by FR Carrick, including a critical review of research articles on its effect or benefit
Source: Chiropr Man Therap. 2020 Jan 28;28:9. doi: 10.1186/s12998-019-0287-2 (PMC6986008; doi:10.1186/s12998-019-0287-2)
Supplement: Supplementary file 1 — Additional file 1: A list of published texts authored or co-authored by FR Carrick obtained on PubMed, Scopus or ResearchGate, shown by study topic, study design and whether full text or not full text documents. [file 12998_2019_287_MOESM1_ESM.docx]

Additional file 1 : A list of published texts authored or co-authored by FR Carrick obtained on PubMed, Scopus or ResearchGate, shown by study topic, study design and whether full text or not full text documents.

| **Publication titles** | **Year of publication** | **Journal** | **Study**  **Topic:**  **Brain**  **Balance**  **Other FN Other non FN** | **Our source** | | | **- Type of article - Full text (Yes/No)** |
| --- | --- | --- | --- | --- | --- | --- | --- |
|  |  |  |  | **PubMed** | **Scopus** | **ResearchGate** |  |
| Colorectal Cancer Risk Awareness and Screening Uptake among Adults in the United Arab Emirates | 2018 | Asian Pac J Cancer Prev 19 (8), 2343-2349 | Other non FN | Yes | Yes | Yes | Survey Yes |
| Burnout and depression among medical residents in the United Arab Emirates: A multicenter study | 2018 | [J Family Med Prim Care.](https://www.ncbi.nlm.nih.gov/pubmed/30090790) 2018 Mar-Apr;7(2):435-441 | Other non FN | Yes | No | No | Survey  Yes |
| The Treatment of Autism Spectrum Disorder With Auditory Neurofeedback: A Randomized Placebo Controlled Trial Using the Mente Autism Device | 2018 | [Front Neurol.](https://www.ncbi.nlm.nih.gov/pubmed/?term=The+Treatment+of+Autism+Spectrum+Disorder+With+Auditory+Neurofeedback%3A+A+Randomized+Placebo+Controlled+Trial+Using+the+Mente+Autism+Device) 2018 Jul 5;9:537 | Brain | Yes | Yes | Yes | Clinical Study  Yes |
| Emergency peripartum hysterectomy in the Dubai health system: A fifteen year experience | 2017 | [Turk J Obstet Gynecol.](https://www.ncbi.nlm.nih.gov/pubmed/?term=Emergency+peripartum+hysterectomy+in+the+Dubai+health+system%3A+A+fifteen+year+experience) 2018 Mar;15(1):1-7 | Other non FN | Yes | Yes | Yes | Hospital records Yes |
| The Federation of Student Islamic Societies Programme to Challenge Mental Health Stigma in Muslim Communities in England: The Fosis Birmingham Study | 2017 | [Psychiatr Danub.](https://www.ncbi.nlm.nih.gov/pubmed/?term=The+Federation+of+Student+Islamic+Societies+Programme+to+Challenge+Mental+Health+Stigma+in+Muslim+Communities+in+England%3A+The+Fosis+Birmingham+Study) 2017 Sep;29(Suppl 3) 512-520 | Other non FN | Yes | Yes | Yes | Clinical study  Yes |
| “A Labour of Love”: A King’s College London Psychiatry society Event to Challenge the Stigma Attached to Mental Health Problems in Post-Natal Women | 2017 | [Psychiatr Danub.](https://www.ncbi.nlm.nih.gov/pubmed/?term=%E2%80%9CA+Labour+of+Love%E2%80%9D%3A+A+king%E2%80%99s+college+London+Psychiatry+society+Event+to+Challenge+the+Stigma+Attached+to+Mental+Health+Problems+in+Post-Natal+Women) 2017 Sep;29(Suppl 3):469-475 | Other non FN | Yes | Yes | Yes | Clinical study  Yes |
| A King’s College London undergraduate Psychiatry Society Event to Challenge the Stigma Attached to Psychological Problems in Healthcare Professionals and students | 2017 | [Psychiatr Danub.](https://www.ncbi.nlm.nih.gov/pubmed/?term=A+king%E2%80%99s+College+London+undergraduate+Psychiatry+Society+Event+Challenge+the+Stigma+Attached+to+Psychological+Problems+in+Healthcare+Professionals+and+students) 2017 Sep;29(Suppl 3):457-463. | Other non FN | Yes | Yes | Yes | Clinical study  Yes |
| Predictors of Postpartum Depression in Dubai, a rapidly Growing Multicultural society in the united Arab Emirates | 2017 | [Psychiatr Danub.](https://www.ncbi.nlm.nih.gov/pubmed/?term=Predictors+Of+Postpartum+Depression+in+Dubai%2C+A+rapidly+Growing+Multicultural+society+in+the+united+Arab+Emirates) 2017 Sep;29(Suppl 3):313-322. | Other non FN | Yes | Yes | No | Survey  Yes |
| The Wounded Healer Film: A London College of Communication Event to Challenge Mental Health Stigma Through the Power of Motion Picture | 2017 | [Psychiatr Danub.](https://www.ncbi.nlm.nih.gov/pubmed/?term=The+Wounded+Healer+Film%3A+A+London+College+of+Communication+Event+to+Challenge+Mental+Health+Stigma+Through+the+Power+of+Motion+Picture) 2017 Sep;29(Suppl 3):307-312 | Other non FN | Yes | Yes | Yes | Clinical study  Yes |
| Harnessing The Power of Film to Combat Mental Health Stigma. A University College London Psychiatry Society Event | 2017 | [Psychiatr Danub.](https://www.ncbi.nlm.nih.gov/pubmed/28953782) 2017 Sep;29(Suppl 3):300-306 | Other non FN | Yes | Yes | Yes | Clinical study  Yes |
| EV1018: The Wounded Healer : An Anti-stigma program target at healthcare professionals and students | 2017 | European Psych vol 41, Supp, April 2017 S735 | Other non FN | No | No | Yes  See comment a. | Conference Paper  No |
| The Federation of Student Islamic Societies Programme To Challenge Mental Health Stigma in Muslim Communities in Ireland: The Fosis Dublin Study | 2017 | [Psychiatr Danub.](https://www.ncbi.nlm.nih.gov/pubmed/?term=The+Federation+of+Student+Islamic+Societies+Programme+To+Challenge+Mental+Health+Stigma+in+Muslim+Communities+in+Ireland%3A+The+Fosis+Dublin+Study) 2017 Sep;29(Suppl 3):279-284 | Other non FN | Yes | Yes | Yes | Clinical study  Yes |
| The performing arts and psychological well-being | 2017 | [Psychiatr Danub.](https://www.ncbi.nlm.nih.gov/pubmed/28953763) 2017 Sep;29(Suppl 3):196-202. | Other non FN | Yes | Yes | Yes | Discussion paper Yes |
| Part II: Muslims Perceptions Of British Cobat (s.i.c!)Troops | 2017 | [Psychiatr Danub.](https://www.ncbi.nlm.nih.gov/pubmed?term=part%20ii%20muslims%20perceptions%20of%20british%20combat%20troops&cmd=correctspelling) 2017 Sep;29(Suppl 3):173-178. | Other non FN | Yes | Yes | Yes | Survey Yes |
| Part I: Muslims, Social Inclusions and the West Exploring Challenges Faced by Stigmatized Groups | 2017 | [Psychiatr Danub.](https://www.ncbi.nlm.nih.gov/pubmed/?term=Part+I%3A+Muslims%2C+Social+Inclusions+and+the+West+Exploring+Challenges+Faced+by+Stigmatized+Groups) 2017 Sep;29(Suppl 3):164-172. | Other non FN | Yes | Yes | No | Discussion paper  Yes |
| Professionalism among multicultural medical Students in the United Arab Emirates | 2017 | [Med Educ Online.](https://www.ncbi.nlm.nih.gov/pubmed/?term=Professionalism+among+multicultural+medical+Students+in+the+United+Arab+Emirates) 2017;22(1):1372669 | Other non FN | Yes | Yes | Yes | Survey  Yes |
| Head-Eye Vestibular Motion Therapy Affects the Mental and Physical Health of Severe Chronic Postconcussion Patients | 2017 | [Front Neurol.](https://www.ncbi.nlm.nih.gov/pubmed/?term=Head-Eye+Vestibular+Motion+Therapy+Affects+the+Mental+and+Physical+Health+of+Severe+Chronic+Postconcussion+Patients) 2017 Aug 22;8:414 | Brain | Yes | Yes | Yes | Clinical study Yes |
| Randomized Controlled Study of a Remote Flipped Classroom Neuro-Otology Curriculum | 2017 | [Front Neurol.](https://www.ncbi.nlm.nih.gov/pubmed/?term=Randomized+Controlled+Study+of+a+Remote+Flipped+Classroom+Neuro-Otology+Curriculum) 2017 Jul 24;8:349. | Other non FN | Yes | Yes | Yes | Clinical Study  Yes |
| Using posturography in a practice-based setting to investigate the effect of saccades in healthy subjects | 2017 | Biomed Sci Instrum 53 ©ISA 2017, 9781945541193/2017 | Posture | No | Yes | Yes | Experimental Study  Yes |
| Decreased symptoms and Increased Function in Post Concussion Syndrome Patients after Coordinated Eye-Head Movement Therapy | 2017 | Conference: Word Congress on brain injury at New Orleans, LA | Brain | No | No | Yes | Unclear No |
| Eye-Movement Training Results in changes in qEEG and NIH Stroke Scale in Subjects Suffering from Acute Middle Cerebral Artery Ischemic Stroke: A Randomized Control Trial | 2016 | [Front Neurol.](https://www.ncbi.nlm.nih.gov/pubmed/26834698) 2016 Jan 22;7:3 | Brain | Yes | Yes | Yes | Clinical study  Yes |
| Cognitive Improvement in a 52-year-old female cognitive decline following a 5-day ReceptorBased treatment. | 2016 | Frontier, conference; international symposium in clinical Neuroscience: Clinical Neuroscience for Optimization of human Function Orlando, United States, 7 Oct – 9 Oct 2016 | Brain | No | No | Yes | Case report No |
| Improvements in balance, vestibular-ocular response, eye-tracking, cognitive processing, pain and range of motion after 5-day intensive rehabilitation program | 2016 | Frontiers,  International Symposium on clinical neuroscience for optimization of human function Orlando, United States, 7 Oct-9 Oct 2016 | Balance | No | No | Yes | Case report No |
| Improvement in symptom severity, cognitive assessment, processing speed, reaction time and visual acuity in a professional hockey player with a prior concussion | 2016 | Frontiers,  Conference : International Symposium on Clinical Neuroscience : Clinical Neuroscience for Optimization of Human Function, Orlando, United States, 7 Oct – 9 Oct | Brain | No | No | Yes | Case report  No |
| Improvement in cognitive assessment, processing speed, and visual acuity in a 10 year old with Pediatric Autoimmune Neuropsychiatric Disorder after Streptococcus infection (PANDAS) | 2016 | Frontiers,  Conference: International Symposium on Clinical Neuroscience: Clinical Neuroscience for Optimization for Human Function, Orlando, United States, 7 Oct-9 Oct | Brain | No | No | Yes | Case report No |
| Improvement in symptoms and processing speed measurements in a professional hockey player with post-concussive syndrome | 2016 | Frontiers, Conference: International Symposium on Clinical Neuroscience: Clinical Neuroscience for Optimization for Human Function, Orlando, United States, 7 Oct-9 Oct | Brain | No | No | Yes | Case report  No |
| Improvement in symptom severity, cognitive assessment, and choice reaction time in a patient with schizophrenia, paranoia, and aggression | 2016 | Frontiers, Conference: International Symposium on Clinical Neuroscience: Clinical Neuroscience for Optimization for Human Function, Orlando, United States, 7 Oct-9 Oct | Brain | No | No | Yes | Case report  No |
| Improvement of symptoms, balance, and gait in a geriatric male, with a 5 day intensive combination of visual, vestibular, proprioceptive, and non-invasive nerve stimulation. | 2016 | Frontiers, Conference: International Symposium on Clinical Neuroscience: Clinical Neuroscience for Optimization for Human Function, Orlando, United States, 7 Oct-9 Oct | Balance | No | No | Yes | Case report  No |
| Improvement in symptom severity, cognitive assessment and processing speed in a patient with vascular dementia | 2016 | Frontiers, Conference: International Symposium on Clinical Neuroscience: Clinical Neuroscience for Optimization for Human Function, Orlando, United States, 7 Oct-9 Oct | Brain | No | No | Yes | Case report  No |
| Improvement in persistent vegetative state of a 4-year-old, 2 years after near-drowning | 2016 | Frontiers, Conference: International Symposium on Clinical Neuroscience: Clinical Neuroscience for Optimization for Human Function, Orlando, United States, 7 Oct-9 Oct | Brain | No | No | Yes | Case report  No |
| Failure to improve post-concussive “brain-fog” and other associated symptoms, despite improvements in reaction time neurocognitive, visual tracking, and vestibular-ocular function following a 5-day intensive rehabilitation program. | 2016 | Frontiers, Conference: International Symposium on Clinical Neuroscience: Clinical Neuroscience for Optimization for Human Function, Orlando, United States, 7 Oct-9 Oct | Brain | No | No | Yes | Case report  No |
| Improvement in muscle tone, coordination, balance, ocular-motor function and communication in a 6-year-old male with developmental delays after 5 days of ReceptorBased treatment | 2016 | Frontiers, Conference: International Symposium on Clinical Neuroscience: Clinical Neuroscience for Optimization for Human Function, Orlando, United States, 7 Oct-9 Oct | Brain | No | No | Yes | Case report  No |
| Improvement in symptom severity, cognitive assessment, and choice reaction time in a college professor with post-concussive syndrome. | 2016 | Frontiers, Conference: International Symposium on Clinical Neuroscience: Clinical Neuroscience for Optimization for Human Function, Orlando, United States, 7 Oct-9 Oct | Brain | No | No | Yes | Case report  No |
| Improvement in symptom severity, processing speed and visual acuity in a patient with a history of multiple concussion and anxiety, with neurological rehabilitation and pulsed magnetic frequency therapy | 2016 | Frontiers, Conference: International Symposium on Clinical Neuroscience: Clinical Neuroscience for Optimization for Human Function, Orlando, United States, 7 Oct-9 Oct | Brain | No | No | Yes | Case report  No |
| Benign Paroxysmal Positional Vertigo and Abdominal Discomfort Relieved by Manipulation of the Sacroiliac Joint | 2016 | Frontiers, Conference: International Symposium on Clinical Neuroscience: Clinical Neuroscience for Optimization for Human Function, Orlando, United States, 7 Oct-9 Oct | Balance | No | No | Yes | Case report  No |
| Accuracy, precision and consistency of robot and therapist generated horizontal head motions for gaze stabilization rehabilitation –Preliminary investigation | 2016 | Biomed Sci Instrum, 52:300-307 | Balance | No | Yes | Yes | Experimental study  Yes |
| Neurological Rehabilitation in Traumatic Brain Injury Relieving Visceral Complaints | 2016 | Frontiers in Neurology 7 DOI: 10.3389/conf.fneur.2016.59.00040 | Brain | No | No | Yes | Unclear  No |
| Improvement in Facial Paralysis with multimodal neurorehabilitation | 2015 | Frontier, Conference: Symposium on clinical neuroscience, TBI and neurodegeration Orlando Florida, United States, 10 Dec - 14 Dec 2015 | Brain | No | No | Yes | Case report  No |
| Challenges facing Medical Residents’ satisfaction in the Middle East: A Report From United Arab Emirates | 2015 | [Teach Learn Med.](https://www.ncbi.nlm.nih.gov/pubmed/26507996) 2015;27(4):387-94 | Other non FN | Yes | Yes | Yes | Survey  Yes |
| Islam, Mental Health and Being a Muslim in the West | 2015 | Psychiatria Danubina, Vol. 27, Suppl. 1, pp 53-59 | Other non FN | Yes | Yes | Yes | Discussion paper  Yes |
| Short- and Long-term effectiveness of a subject’s specific novel brain and vestibular rehabilitation treatment modality in combat veterans suffering from PTSD | 2015 | [Front Public Health.](https://www.ncbi.nlm.nih.gov/pubmed/?term=Short-+and+Long-term+effectiveness+of+a+subject%E2%80%99s+specific+novel+brain+and+vestibular+rehabilitation+treatment+modality+in+combat+veterans+suffering+from+PTSD) 2015 Jun 1;3:151. | Brain | Yes | No | Yes | Clinical study  Yes |
| Effect of tone-based sound stimulation on balance performance of normal subjects: Preliminary investigation | 2015 | [Biomed Sci Instrum.](https://www.ncbi.nlm.nih.gov/pubmed/?term=Effect+of+tone-based+sound+stimulation+on+balance+performance+of+normal+subjets%3A+Preliminary+investigation) 2015;51:54-61 | Balance | Yes | Yes | Yes | Experimental Study  Yes |
| Changes in saccadic eye movements produced by novel brain and vestibular rehabilitation therapy | 2015 | [Biomed Sci Instrum.](https://www.ncbi.nlm.nih.gov/pubmed/?term=Changes+in+saccadic+eye+movements+produced+by+novel+brain+and+vestibular+rehabilitation+therapy) 2015;51:9-16. | Brain | Yes | Yes | Yes | Clinical Study  Yes |
| Improvement in mild traumatic brain injury through multimodal neurorehabilitation | 2015 | Frontiers, Conference: International Symposium on Clinical Neuroscience: TBI and Neurodegeneration, Orlando, Florida, United States, 10 Dec – 14 Dec 2015 | Brain | No | No | Yes | Case report  No |
| Teenage Male with Mild Traumatic Brain Injury Improved Through a Comprehensive Neurorehabilitation Regimen | 2015 | Frontiers, Conference: International Symposium on Clinical Neuroscience: TBI and Neurodegeneration, Orlando, Florida, United States, 10 Dec – 14 Dec 2015 | Brain | No | No | Yes | Case report  No |
| Anismus Treated Through Multimodal Neurorehabilitation | 2015 | Frontiers, Conference: International Symposium on Clinical Neuroscience: TBI and Neurodegeneration, Orlando, Florida, United States 10 Dec – 14 Dec 2015 | Other FN | No | No | Yes | Case report  No |
| Multimodal Neurorehabilitation in Patient with Spastic Cerebral Palsy | 2015 | Frontiers, Conference: International Symposium on Clinical Neuroscience: TBI and Neurodegeneration, Orlando, Florida, United States, 10 Dec – 14 Dec 2015 | Brain | No | No | Yes | Case report  No |
| Repetitive Peripheral Somatosensory stimulation improves transverse myelitis | 2015 | Frontiers, Conference: International Symposium on Clinical Neuroscience: TBI and Neurodegeneration, Orlando, Florida, United States, 10 Dec – 14 Dec 2015 | Other FN | No | No | Yes | Case report  No |
| Multimodal neurorehabilitation improves patient with progressive aphasia | 2015 | Frontiers, Conference: International Symposium on Clinical Neuroscience: TBI and Neurodegeneration, Orlando, Florida, United States, 10 Dec – 14 Dec 2015 | Brain | No | No | Yes | Case report  No |
| Vasovagal syncope improves after multimodal neurorehabilitation | 2015 | Frontiers, Conference: International Symposium on Clinical Neuroscience: TBI and Neurodegeneration, Orlando, Florida, United States, 10 Dec – 14 Dec 2015 | Brain | No | No | Yes | Case report  No |
| Vestibular stimulation and its impact on stability and balance for a person with multisystem atrophy-like syndrome : case study | 2015 | Frontiers, Conference: International Symposium on Clinical Neuroscience: TBI and Neurodegeneration, Orlando, Florida, United States, 10 Dec – 14 Dec 2015 | Balance | No | No | Yes | Case report  No |
| Multimodal neurorehabilitation improves vertigo and diplopia | 2015 | Frontiers, Conference: International Symposium on Clinical Neuroscience: TBI and Neurodegeneration, Orlando, Florida, United States, 10 Dec – 14 Dec 2015 | Brain | No | No | Yes | Case report  No |
| Balance and headache in patient with mild traumatic brain injury improve with multi-modal neurorehabilitation | 2015 | Frontiers, Conference: International Symposium on Clinical Neuroscience: TBI and Neurodegeneration, Orlando, Florida, United States, 10 Dec – 14 Dec 2015 | Brain | No | No | Yes | Case report  No |
| Neurorehabiliation (s.i.c!) improves diploplia and nystagmus following traumatic brain injury | 2015 | Frontiers, Conference: International Symposium on Clinical Neuroscience: TBI and Neurodegeneration, Orlando, Florida, United States, 10 Dec – 14 Dec 2015 | Brain | No | No | Yes | Case report  No |
| Mild traumatic brain injury improved through an integrated neurorehabilitation approach | 2015 | Frontiers, Conference: International Symposium on Clinical Neuroscience: TBI and Neurodegeneration, Orlando, Florida, United States, 10 Dec – 14 Dec 2015 | Brain | No | No | Yes | Case report  No |
| Spastic paraplegia improves with multimodal neurorehabilitation | 2015 | Frontiers, Conference: International Symposium on Clinical Neuroscience: TBI and Neurodegeneration, Orlando, Florida, United States, 10 Dec – 14 Dec 2015 | Brain | No | No | Yes | Case report  No |
| Treatment of post concussion syndrome (PCS) with hemi-neglect following a mild traumatic brain injury (mTBI) in a hockey player : case study | 2015 | Frontiers, Conference: International Symposium on Clinical Neuroscience: TBI and Neurodegeneration, Orlando, Florida, United States,10 Dec – 14 Dec 2015Brain | Brain | No | No | Yes | Case report  No |
| [Functional improvements in a patient with traumatic brain injury and dystonia following integrated neurorehabilitation](https://www.researchgate.net/publication/283566261_FUNCTIONAL_IMPROVEMENTS_IN_A_PATIENT_WITH_TRAUMATIC_BRAIN_INJURY_AND_DYSTONIA_FOLLOWING_INTEGRATED_NEUROREHABILITATION) | 2015 | Frontiers, Conference: International Symposium on Clinical Neuroscience: TBI and Neurodegeneration, Orlando, Florida, United States, 10 Dec – 14 Dec 2015 | Brain | No | No | Yes | Case report  No |
| Post concussion vertigo and headaches improve after multimodal neurorehabilitation | 2015 | Frontiers, Conference: International Symposium on Clinical Neuroscience: TBI and Neurodegeneration, Orlando, Florida, United States, 10 Dec – 14 Dec 2015 | Brain | No | No | Yes | Case report  No |
| Multimodal neurorehabilitation in the treatment of mild traumatic brain injury | 2015 | Frontiers, Conference: International Symposium on Clinical Neuroscience: TBI and Neurodegeneration, Orlando, Florida, United States, 10 Dec – 14 Dec 2015 | Brain | No | No | Yes | Case report  No |
| Multimodal neurorehabilitation improves balance in patient with mild traumatic brain injury | 2015 | Frontiers, Conference: International Symposium on Clinical Neuroscience: TBI and Neurodegeneration, Orlando, Florida, United States, 10 Dec – 14 Dec 2015 | Brain | No | No | Yes | Case report  No |
| Multimodal neurorehabilitation improves teenage female with mild traumatic brain injury | 2015 | Frontiers, Conference: International Symposium on Clinical Neuroscience: TBI and Neurodegeneration, Orlando, Florida, United States, 10 Dec – 14 Dec 2015 | Brain | No | No | Yes | Case report  No |
| Multimodal neurorehabilitation in young boy with complex regional pain syndrome | 2015 | Frontiers, Conference: International Symposium on Clinical Neuroscience: TBI and Neurodegeneration, Orlando, Florida, United States, 10 Dec – 14 Dec 2015 | Other FN | No | No | Yes | Case report  No |
| Ataxia improved through application of multimodal neurorehabilitation approach | 2015 | Frontiers, Conference: International Symposium on Clinical Neuroscience: TBI and Neurodegeneration, Orlando, Florida, United States, 10 Dec – 14 Dec 2015 | Balance | No | No | Yes | Case report  No |
| Multimodal neurorehabilitation on patient with stiff man syndrome | 2015 | Frontiers, Conference: International Symposium on Clinical Neuroscience: TBI and Neurodegeneration, Orlando, Florida, United States, 10 Dec – 14 Dec 2015 | Other FN | No | No | Yes | Case report No |
| Improvements in a patient with mild traumatic brain injury and dyslexia following multimodal neurorehabilitation | 2015 | Frontiers, Conference: International Symposium on Clinical Neuroscience: TBI and Neurodegeneration, Orlando, Florida, United States, 10 Dec – 14 Dec 2015 | Brain | No | No | Yes | Case report  No |
| Resolution of blepherospasm through multimodal neurorehabiliation (s.i.c!) | 2015 | Frontiers, Conference: International Symposium on Clinical Neuroscience: TBI and Neurodegeneration, Orlando, Florida, United States, 10 Dec – 14 Dec 2015 | Brain | No | No | Yes | Case report  No |
| Neurorehabilitation for patient with dysautonomia | 2015 | Frontiers, Conference: International Symposium on Clinical Neuroscience: TBI and Neurodegeneration, Orlando, Florida, United States, 10 Dec – 14 Dec 2015 | Other FN | No | No | Yes | Case report  No |
| Vertigo improved with multimodal neurorehabilitation program | 2015 | Frontiers, Conference: International Symposium on Clinical Neuroscience: TBI and Neurodegeneration, Orlando, Florida, United States, 10 Dec – 14 Dec 2015 | Balance | No | No | Yes | Case report  No |
| Integrative neurorehabilitation improves post-concussion syndrome | 2015 | Frontiers, Conference: International Symposium on Clinical Neuroscience: TBI and Neurodegeneration, Orlando, Florida, United States, 10 Dec – 14 Dec 2015 | Brain | No | No | Yes | Case report  No |
| Recalibration of oculomotor and cerebellar activity to reduce low back pain in a professional tennis player | 2015 | Frontiers, Conference: International Symposium on Clinical Neuroscience: TBI and Neurodegeneration, Orlando, Florida, United States, 10 Dec – 14 Dec 2015 | Brain | No | No | Yes | Case report  No |
| Unilateral Arm and Leg Tremor Improve from Multimodal Neurorehabilitation | 2015 | Frontiers, Conference: International Symposium on Clinical Neuroscience: TBI and Neurodegeneration, Orlando, Florida, United States, 10 Dec – 14 Dec 2015 | Brain | No | No | Yes | Case report  No |
| Neurorehabilitation improves Patient with repititive (s.i.c!) Traumatic Brain Injuries | 2015 | Frontiers, Conference: International Symposium on Clinical Neuroscience: TBI and Neurodegeneration, Orlando, Florida, United States, 10 Dec – 14 Dec 2015 | Brain | No | No | Yes | Case report  No |
| Post-concussion dizziness improves from multimodal neurorehabilitation | 2015 | Frontiers, Conference: International Symposium on Clinical Neuroscience: TBI and Neurodegeneration, Orlando, Florida, United States, 10 Dec – 14 Dec 2015 | Brain | No | No | Yes | Case report  No |
| Improvements of memory loss and digestion following multimodal neurorehabilitation strategies | 2015 | Frontiers, Conference: International Symposium on Clinical Neuroscience: TBI and Neurodegeneration, Orlando, Florida, United States, 10 Dec – 14 Dec 2015 | Brain | No | No | Yes | Case report  No |
| Multimodal neurorehabilitation improve (s.i.c!) mild traumatic brain injury | 2015 | Frontiers, Conference: International Symposium on Clinical Neuroscience: TBI and Neurodegeneration, Orlando, Florida, United States, 10 Dec – 14 Dec 2015 | Brain | No | No | Yes | Case report  No |
| Abstract: case report of multiple syncopic events with resultant concussions, vertigo, dysautonomia, migraines, aphasia and paralysis in a 28 year-old female after traumatic brain injury | 2015 | Frontiers, International Symposium on Clinical Neuroscience: TBI and Neurodegeneration, Orlando, Florida, United States, 10 Dec - 14 Dec, 2015. | Brain | No | No | Yes | Case report  No |
| Return to play for a hockey player with persistent post concussion syndrome and hemi-neglect following traumatic brain injury (TBI): a case study | 2015 | Frontiers, Conference: International Symposium on Clinical Neuroscience: TBI and Neurodegeneration, Orlando, Florida, United States, 10 Dec – 14 Dec 2015 | Brain | No | No | Yes | Case report  No |
| Treatment of cervical dystonia through multimodal neurorehabilitation | 2015 | Frontiers, Conference: International Symposium on Clinical Neuroscience: TBI and Neurodegeneration, Orlando, Florida, United States, 10 Dec – 14 Dec 2015 | Other FN | No | No | Yes | Case report  No |
| Abstract T P121 : Combinations of fast and slow eye movement strategies improve clinical outcomes and EEG activity in stroke patients, a randomized controlled trial | 2015 | Stroke, vol.46, no.supp1 | Brain | No | No | Yes | Clinical study  No |
| Heart rate variability for assessing comatose patients with different Glasgow Come Scale scores | 2015 | Premio Anual de Salud 2014 | Brain | No | No | Yes | Award for a study Yes |
| Evaluation of the effectiveness of a novel brain and vestibular rehabilitation treatment modality in PTSD patients who have suffered combat-related traumatic brain injuries | 2015 | [Front Public Health](https://www.ncbi.nlm.nih.gov/pmc/articles/PMC4316606/). 2015; 3: 15. | Brain | Yes | Yes | Yes | Clinical Study  Yes |
| Between-subjects differences of within-subject variability in repeated balance measures: Consequences on the minimum detectable change | 2015 | Gait & Posture 41 (2015) 136-140 | Balance | Yes | Yes | Yes | Experimental study  Yes |
| In-situ verification of accuracy, precision and resolution of force and balance platforms | 2014 | Biomed Sci Instrum 2014; 50:171-8 | Balance | Yes | Yes | Yes | Unclear  No |
| Letter to the editor:  On “Validity and reliability of the Nintendo Wii Balance Board for assessment of standing balance” by R.A. Clark et al. [Gait & Posture 31 (2010) 307–310]: are the conclusions stated by the authors justified? | 2014 | Gait & Posture 39 (2014) 1149 – 1161 | Balance | Yes | Yes | Yes | Letter to editor  Yes |
| On "Comparison of a laboratory grade force platform with a Nintendo Wii Balance Board on measurement  of postural control in single-leg stance balance tasks" by Huurnink, A., et al.  [J. Biomech 46(7) (2013) 1392]: Are the conclusions stated by the authors justified? | 2014 | [J Biomech.](https://www.ncbi.nlm.nih.gov/pubmed/24359674) 2014 Feb 7;47(3):759-60. | Balance | Yes | Yes | Yes | Letter to editor  Yes |
| Zolpidem arousing effect in persistent vegetative state  patients: autonomic, EEG and behavioral assessment. | 2014 | [Curr Pharm Des.](https://www.ncbi.nlm.nih.gov/pubmed/24025063) 2014;20(26):4185-202. | Brain | Yes | Yes | Yes | Clinical study  Yes |
| Zolpidem induces paradoxical metabolic and vascular changes in a patient with PVS | 2013 | [Brain Inj.](https://www.ncbi.nlm.nih.gov/pubmed/23924270) 2013; 27(11):1320-9. | Brain | Yes | Yes | Yes | Case report  Yes |
| Unusual Brainstem Twisting Revealed by MRI tractography in a Patient Who Survived a severe Traumatic Upper Spinal Dislocation | 2013 | Funct Neurol Rehabil Ergon 2013; 3(4):421-422 | Brain | No | No | Yes  See  Comment b | Case report  Yes |
| The Treatment of Persistent Imbalance in patient with Traumatic Brain Injury Using A functional Neurological Approach | 2013 | Funct Neurol Rehabil Ergon 2013; 3(4): 423-429 | Brain | No | No | No  See  Comment c | Case report  Yes |
| Quantitative resting EEG in the autistic spectrum disorder | 2013 | International Journal of Child health and human development 6(4):511 | Brain | No | No | Yes  See comment d | Unclear  No |
| Frequency content of standard posturographic measures | 2013 | Biomed Sci Instrum 2013; 49:48-53 | Posture | Yes | Yes | Yes | Experimental study  Yes |
| Frequency content of standard posturographic measures | 2013 | 50^th^ Annual Rocky Mountain Bioengineering Symposium and 50^th^ International ISA Biomedical Sciences Instrumentation Symposium 2013, 493, pp. 49-54 | Posture | No | Yes | No | Experimental study No |
| The Effect of Off Vertical Axis and Multiplanar Vestibular Rotational Stimulation on Balance Stability and Limits of Stability | 2013 | Funct Neurol Rehab Ergon 2013;3(2-3):342-360 | Balance | No | No | Yes | Experimental study  Yes |
| Heart rate variability for assessing comatose patients with different Glasgow Coma Scale scores | 2013 | [Clin Neurophysiol.](https://www.ncbi.nlm.nih.gov/pubmed/23063293)   2013 Mar;124(3): 589-97 | Brain | Yes | Yes | Yes | Clinical study  Yes |
| Vegetative state is a pejorative term | 2012 | Neuro Rehabilitation 31 (2012) 345-347 | Brain | Yes | Yes | Yes | Discussion paper  Yes |
| Neuroimaging Findings in Non-fatal Central Transtentorial Herniation | 2012 | Funct Neurol Rehabil Ergon 2012; 2(2) 127-131 | Brain | No | No | Yes  See comment b. | Case report  Yes |
| Neuroimaging Findings in Non-fatal Central Transtentorial Herniation | 2012 | Conference Unknow | Brain | No | No | yes | Conference paper |
| Neuroimaging-Based Assessment of Default Network Connectivity in Brain Damaged Patients | 2012 | Conference: 2^nd^ International Symposium on Disorders of Consciousness, Havana, Cuba, December 6-8 2011. | Brain | No | No | Yes | Case series  No |
| Neuroimaging-Based Assessment of Default Network Connectivity in Brain Damaged Patients | 2012 | Journal unknown | Brain | No | No | Yes | Case series  No |
| Bilateral N20 absence in post-anoxic coma: Do you pay attention? | 2012 | [Clin Neurophysiol.](https://www.ncbi.nlm.nih.gov/pubmed/22209660)  2012 Jul;123(7):1264-6 | Brain | Yes | Yes | Yes | Editorial  Yes |
| qEEG may increase the reliability of diagnostic and prognostic procedures in cerebral arterial gas embolism | 2012 | [Clin Neurophysiol.](https://www.ncbi.nlm.nih.gov/pubmed/21784702)  2012 Feb;123(2):225-6 | Brain | Yes | Yes | Yes | Editorial  Yes |
| Learning effect of standing on foam during posturographic testing – preliminary findings | 2012 | [Biomed Sci Instrum.](https://www.ncbi.nlm.nih.gov/pubmed/22846302)  2012;48:332-9 | Posture | Yes | Yes | Yes | Experimental study Yes |
| Learning effect of standing on foam during posturographic testing – Preliminary findings | 2012 | 49^th^ Annual Rocky Mountain Bioengineering Symposium and 49^th^ International ISA Biomedical Sciences Instrumentation Symposium, pp. 219-226 | Posture | No | Yes | No | Experimental study No |
| Blood pressure patterns after brain death | 2011 | Neurology July 26, 2011 77:399-401 | Other non FN | No | No | Yes | Case report  No |
| The effects of whole body rotations in the pitch and yaw planes on postural stability | 2011 | Funct Neurol Rehabil Ergon 2011; 1(2): 167-179 | Posture | No | No | No  See comment e | Experimental study yes |
| The effect of hemisphere specific remediation strategies on the academic performance outcome of children with ADD/ADHD | 2010 | Int J Adolesc Med Health 2010; 22(2):275-283 | Brain | Yes | Yes | Yes | Clinical Study  Yes |
| Effect of contralateral extremity manipulation on brain function | 2010 | Int J Disabil Hum Dev 2010;9(4):269-273 | Brain | No | Yes | Yes | Clinical study  Yes |
| 281: Cognitive changes in ADHD Children after 12 week Postural rehabilitation program | 2010 | Posters/Parkinsonism and related Disorders 16S1 (2010) S11 – S86 | Brain | No | No | Yes  See comment b | Clinical study  No |
| 068: A relationship between postural and cognitive abilities in ADHD | 2010 | Posters/Parkinsonism and related Disorders 16S1 (2010) S11 – S86 | Brain | No | No | Yes | Clinical study  No |
| 128:Disorder of Movement And Motor Function Do Not Affect Consciousness but Facilitate Cognitive and Motor Plasticity | 2010 | Posters/Parkinsonism and related Disorders 16S1 (2010) S11 – S86 | Brain | No | No | Yes | Clinical study  No |
| Repeatability of Posturographic Measures of the mCTSIB balance tests – a preliminary investigation | 2008 | [Biomed Sci Instrum.](https://www.ncbi.nlm.nih.gov/pubmed/19141890) 2008;44:41-6. | Balance | Yes | Yes | Yes  See comment f | Experimental study  Yes |
| Repeatability of Posturographic Measures of the mCTSIB balance tests – a preliminary investigation | 2008 | Technical Papers of ISA, pp. 20-25 | Balance | No | Yes | No  See comment f | Experimental study  Yes |
| Posturographic testing motor learning predictability in gymnasts | 2007 | [Disabil Rehabil.](https://www.ncbi.nlm.nih.gov/pubmed/17852265) 2007 Dec 30;29(24):1881-9 | Balance | Yes | Yes | Yes | Clinical Study  Yes |
| Posturographic Changes Associated with Music Listening | 2007 | [J Altern Complement Med.](https://www.ncbi.nlm.nih.gov/pubmed/17604555) 2007 Jun;13(5):519-26. | Balance | Yes | Yes | Yes | Experimental study  Yes |
| Balance impairment and music therapy: posturographic changes associated with Nolwenn effect | 2006 | Eura Medicophys 3(2): 82-84. | Balance | No | No | No  See comment g | Conference Paper No |
| The Treatment of Cervical Dystonia by Manipulation of the Cervical Spine: a Study of Brain Hemisphericity, Patient Attributes, And dystonia Characteristics | 2001 | The International Journal of Applied Kinesiology and Kinesiologic Medicine 2001 issue 10 | Other non FN | No | No | No  See comment g | Clinical study No |
| Changes in Brain Function After Manipulation of the Cervical Spine | 1997 | J Manipulative Physiol Ther. 1997 Oct; 20(8)529-45 | Brain | Yes | Yes | Yes | Experimental study  Yes |
| Neurophysiological implications in learning | 1996 | Walden University | Brain | No | No | Yes | Thesis  Yes |
| Cervical Radiculopathy: The diagnosis and treatment of Pathomechanics in the cervical spine | 1983 | J Manipulative Physiol Ther. 1983 Sep;6(3):129-37 | Other non FN | Yes | Yes | Yes | Clinical study  Yes |
| Treatment of Pathomechanics of the Lumbar Spine by Manipulation | 1981 | J Manipulative Physiol Ther. 1981 Dec; 4: 173 – 78 | Other non FN | No | No | No  See comment g | Clinical study Yes |

Comments :

1. On ResearchGate this article can be retrieved but this version does not include FR Carrick as one of the authors.
2. This title was reported twice on ResearchGate.
3. This title was not listed anywhere but the article appeared under another title.
4. On ResearchGate when clicking on this title a different article appears on which FR Carrick is not one of the co-authors.
5. This article was found in the journal Functional Neurology and Rehabilitation Ergonomics.
6. This article appears to be reported twice on Scopus.
7. This article was found on Carrick Institute website, <https://carrickinstitute.com/research/> (access 22/04/2019)
