# Supplementary material for: Unravelling Functional Neurology: an overview of all published documents by FR Carrick, including a critical review of research articles on its effect or benefit
Source: Chiropr Man Therap. 2020 Jan 28;28:9. doi: 10.1186/s12998-019-0287-2 (PMC6986008; doi:10.1186/s12998-019-0287-2)
Supplement: Supplementary file 2 — Additional file 2: a: The number of published full text documents authored or co-authored by FR Carrick shown by type of publication (N = 53).b: The number of published non full text documents authored or co-authored by FR Carrick shown by type of publication (N = 68). [file 12998_2019_287_MOESM2_ESM.docx]

Additional file 2a : The number of published *full text documents* authored or co-authored by FR Carrick shown by type of publication (N=53).

| **Topics** | **Full Text Documents** | | | | | |
| --- | --- | --- | --- | --- | --- | --- |
|  | **N** | **Case report/ Case series** | **Discussion**  **papers /  Letters to editor/ Editorial/ Thesis** | **Survey/ Hospital records** | **Clinical Studies** | **Experimental studies** |
| **Brain** | 20 | 4 | 5 | 0 | 10 | 1 |
| **Balance/ Posture** | 14 | 0 | 2 | 0 | 1 | 11 |
| **Other FN** | 0 | 0 | 0 | 0 | 0 | 0 |
| **Other non FN** | 19 | 0 | 3 | 7 | 9 | 0 |
| ***Total*** | ***53*** | ***4*** | ***10*** | ***7*** | ***20*** | ***12*** |

| **Topics** | **Non Full Text Documents (abstracts only)** | | | | | | |
| --- | --- | --- | --- | --- | --- | --- | --- |
|  | **N** | **Case report/ Case series** | **Discussion**  **papers /  Letters to editor/ Conference papers** | **Survey/ Hospital records** | **Clinical Studies** | **Experimental studies** | **Unclear** |
| **Brain** | 50 | 41 | 1 | 0 | 5 | 0 | 3 |
| **Balance/ Posture** | 10 | 6 | 1 | 0 | 0 | 2 | 1 |
| **Other FN** | 6 | 6 | 0 | 0 | 0 | 0 | 0 |
| **Other non FN** | 2 | 1 | 1 | 0 | 0 | 0 | 0 |
| ***Total*** | ***68*** | ***54*** | ***3*** | ***0*** | ***5*** | ***2*** | ***4*** |

Additional file 2b : The number of published *non full text documents* authored or co-authored by FR Carrick shown by type of publication (N=68).
